# Is valve choice the most important determinant of paravalvular leak following TAVI?

**DOI:** 10.1186/1749-8090-8-S1-O319

**Published:** 2013-09-11

**Authors:** Katie E O'Sullivan, Aideen Gough, Ricardo Segurado, Mitchel Barry, Declan Sugrue, John Hurley

**Affiliations:** 1 Department of Cardiothoracic Surgery, Mater Hospital, Dublin, Ireland; 2 Centre for Support and Training in Analysis and Research (CSTAR), University College Dublin, Ireland; 3 Department of General Surgery, Mater Hospital, Dublin, Ireland; 4 Department of Cardiology, Mater Hospital, Dublin, Ireland

## Background

Paravalvular regurgitation (PVR) following transcatheter aortic valve implantation (TAVI) is associated with poor survival. The two main valve delivery systems used to date differ in a variety of ways. The primary objective of this study was to perform a systematic review and meta-analysis of studies identifying PVR in patients post TAVI to firstly identify the overall incidence and secondly differentiate between valve types. The secondary objective was to identify additional factors predisposing to PVR to determine whether valve choice is the major determinant of PVR.

## Methods

A systematic review and meta-analysis to identify PVR rates was performed. We also sought to examine other factors predisposing to PVR. A total of 19 studies were identified. For post procedure at six months and one year; 7,652, 3,340 and 3,673 patients were included in the analysis of incidence of PVR. For valve-specific analysis a total of 5910 patients were identified from 9 studies.

## Results

The pooled analysis of PVR incidence was 8.21, 10.2 and 10.98% post procedure, at 6 months and 1 year. Formally comparing the CV and ES valve leakage rates by mixed-effects meta-regression with a fixed-effect moderator variable for valve type (MCV or ES), suggested a statistically significant difference in leakage rate between the two valve types (p = 0.0002). MCV was associated with a higher PVR rate of 15.75% [95% CI 12.48-19.32] compared with ES 3.93% [95% CI 1.05-8.38]. Additional modifiable factors predisposing to PVR are valve position, prosthesis-annulus discongruence. Non-modifiable factors are LVOT-AO angle and aortic valve calcification.

## Conclusion

Unfavorable anatomic and pathological factors as well as valve choice have an impact on rates of PVR. Limited by the current level of comparable evidence examining causes of PVR post TAVI, valve choice appears to be the main determinant.

